# Struggling with COVID-19 in Adult Inborn Errors of Immunity Patients: A Case Series of Combination Therapy and Multiple Lines of Therapy for Selected Patients

**DOI:** 10.3390/life13071530

**Published:** 2023-07-08

**Authors:** Patrick Bez, Giancarlo D’ippolito, Carla Maria Deiana, Renato Finco Gambier, Andrea Pica, Giulia Costanzo, Giulia Garzi, Riccardo Scarpa, Nicholas Landini, Francesco Cinetto, Davide Firinu, Cinzia Milito

**Affiliations:** 1 Rare Diseases Referral Center, Internal Medicine 1, Ca’ Foncello Hospital-AULSS2 Marca Trevigiana, 31100 Treviso, Italy; patrick.bez@studenti.unipd.it (P.B.); renato.fincogambier@aulss2.veneto.it (R.F.G.); riccardo.scarpa@aulss2.veneto.it (R.S.); francesco.cinetto@unipd.it (F.C.); 2 Department of Medicine-DIMED, University of Padova, 35122 Padua, Italy; 3 Department of Molecular Medicine, Sapienza University of Rome, 00161 Rome, Italy; giancarlo.dippolito@uniroma1.it (G.D.); andrea.pica@uniroma1.it (A.P.); giulia.garzi@uniroma1.it (G.G.); cinzia.milito@uniroma1.it (C.M.); 4 Department of Medical Sciences and Public Health, University of Cagliari, 09100 Cagliari, Italy; g.costanzo@aoucagliari.it (G.C.); davide.firinu@unica.it (D.F.); 5 Department of Radiological, Oncological and Anatomopathological Sciences, Sapienza University of Rome, Policlinico Umberto I Hospital, 00161 Rome, Italy; nicholas.landini@uniroma1.it

**Keywords:** COVID-19, SARS-CoV-2, IEIs, antivirals, monoclonal antibodies, CVID

## Abstract

Background: The SARS-CoV-2 infection is now a part of the everyday lives of immunocompromised patients, but the choice of treatment and the time of viral clearance can often be complex, exposing patients to possible complications. The role of the available antiviral and monoclonal therapies is a matter of debate, as are their effectiveness and potential related adverse effects. To date, in the literature, the amount of data on the use of combination therapies and on the multiple lines of anti-SARS-CoV-2 therapy available to the general population and especially to inborn error of immunity (IEI) patients is small. Methods: Here, we report a case series of five adult IEI patients managed as inpatients at three Italian IEI referral centers (Rome, Treviso, and Cagliari) treated with combination therapy or multiple therapeutic lines for SARS-CoV-2 infection, such as monoclonal antibodies (mAbs), antivirals, convalescent plasma (CP), mAbs plus antiviral, and CP combined with antiviral. Results: This study may support the use of combination therapy against SARS-CoV-2 in complicated IEI patients with predominant antibody deficiency and impaired vaccine response.

## 1. Introduction

**Figure 1:**
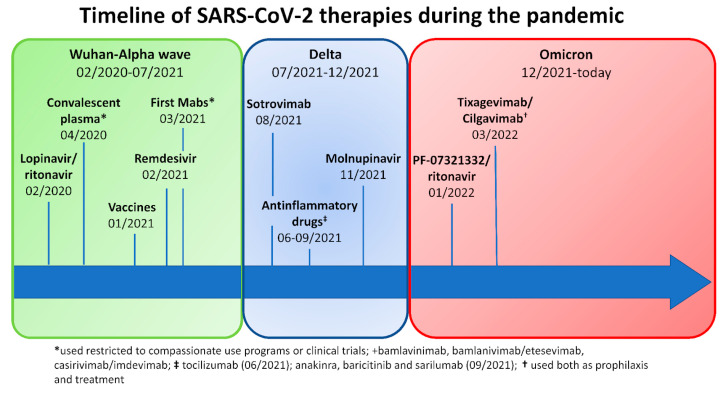
Timeline of SARS-CoV-2 treatments available during the pandemic in Italy.

In Italy, since the start of the pandemic, more than 25 million people have contracted SARS-CoV-2 infection, and over 180,000 have died from COVID-19 disease (https://www.epicentro.iss.it/coronavirus/sars-cov-2-dashboard; last access: 16 February 2023). The severity of SARS-CoV-2 infection ranges from asymptomatic/mild to life-threatening [1]. Since the outbreak of SARS-CoV-2 in 2019, our knowledge of the virus has improved, and this has led to the development of specific prophylactic and therapeutic strategies, such as vaccination, antivirals (e.g., remdesivir and nirmatrelvir/ritonavir), and monoclonal antibodies (mAbs) (e.g., bamlanvimab, sotrovimab, and casivimab/indevimab) [2,3]. Conversely, new variants of concern (VOCs) have been emerging with different virulence and potential resistance to available treatment strategies. Despite the progress, the management of SARS-CoV-2 can still be challenging in patients with inborn errors of immunity (IEI), lymphoproliferative disease, and lymphomas, as these patients have a higher risk of hospitalization and mortality than the general population [4,5,6,7,8]. An extensive review of the literature was recently published [9]. Moreover, we have recently shown that common variable immunodeficiency (CVID) patients with longer positivity, chronic lung disease, and a complicated phenotype according to Chapel’s classification are more prone to bacterial superinfection and death than the general population [10]. To better underline the possible management challenges of COVID-19 in complicated IEI patients, we report a case series of five patients followed at three Italian referral centers who were treated during the same infectious episode with multiple lines of SARS-CoV-2 specific treatments including monoclonal antibodies (mAbs), antiviral drugs, and/or convalescent plasma (CP). Figure 1 recapitulates the key events of the COVID-19 pandemic and the availability of treatments over time.

## 2. Material and Methods

### Patient Cohort and Clinical Data

Data were collected retrospectively in three Italian referral centers (Department of Molecular Medicine, Sapienza, Rome; Rare Diseases Referral Center, Internal Medicine 1, Ca’ Foncello Hospital, Treviso; Department of Medical Sciences and Public Health, Cagliari). IEI patients with documented SARS-CoV-2 infection (RT-PCR-confirmed) who received more than 1 treatment for COVID-19 were included. The demographic data, COVID-19 presentation, treatment details, and outcomes were recorded.

The study has been approved by the Ethical Committee of the Sapienza University of Rome. Informed consent was waived by the Institutional Review Board due to the retrospective observational nature of the study and because participant data were encrypted before analysis. The study was performed in accordance with the Good Clinical Practice guidelines, the International Conference on Harmonization guidelines, and the most recent version of the Declaration of Helsinki.

## 3. Results

### Patient Cohort

PATIENT 1.

A 50-year-old woman with a 10-year history of rheumatoid arthritis (RA) who is refractory to disease-modifying antirheumatic drugs (DMARDs) and has been treated with anti-CD20 monoclonal antibody rituximab (RTX) since 2019. In February 2020, during the administration of a 4-week course of RTX, the patient presented to the Emergency Department (ED) with fever, cough, and flu-like symptoms. The nasopharyngeal swab (NPS) tested positive for SARS-CoV-2 by RT-PCR. A CT scan confirmed COVID-19 bilateral pneumonia. Based on the preliminary data on COVID-19 treatment available at that time [11,12], she was treated with hydroxychloroquine and intravenous methylprednisolone 0.5 mg/kg for 10 days with little improvement. During the following weeks, she continued to have fatigue, a mild cough, and dyspnea without the need for supplemental oxygen. NPS for SARS-CoV-2 by RT-PCR persisted as positive, and serological testing did not detect IgG or IgM antibodies to SARS-CoV-2. Of note, the patient presented with marked hypogammaglobulinemia and complete depletion of peripheral blood CD19+ B cells. Therefore, remdesivir was initiated (5-day course at a dose of 200 mg intravenously on the first day and 100 mg on the remaining days) in the context of a compassionate use program approved by the EMA and the local institution. After treatment, the patient’s conditions improved, and the chest CT performed on day 41 showed an initial resolution of the pneumonia. After 60 days of hospitalization, she was discharged with a still-positive NPS. Repeated serologic testing did not detect IgG antibodies against SARS-CoV2. Ten days later, the patient presented again to the ED with a fever, cough, and respiratory failure. A chest X-ray showed bilateral lung opacities. Due to the increasing oxygen supplementation needed and the protracted course of COVID-19, as well as the lack of other available treatments, COVID-19 CP (neutralizing titer ≥1:160) was administered in a compassionate use program. The infusion was well tolerated, and within 24 h the patient’s condition started to improve. The hospitalization was complicated by recurrent bacterial superinfections. The patient was discharged with a still-positive NPS. On day 192, the patient was admitted again due to the typical clinical and radiological picture of COVID-19 pneumonia and was treated with COVID-19 CP (600 mL) in combination with a course of remdesivir for 5 days (200 mg intravenously on the first day and 100 mg on the remaining days). Her fever disappeared within 24 h, and the oxygen was weaned off in a few days. She was discharged after one week. After 260 days of positivity, she tested negative for SARS-CoV-2. The chest CT scan showed the resolution of SARS-CoV-2-related pneumonia. Afterwards, the patient was referred to our outpatient clinic. The medical records showed a history of recurrent bacterial infections and hypogammaglobulinemia before the RA diagnosis, consistent with a delayed diagnosis of CVID. Thus, IgRT was started.

This case shows an extremely prolonged COVID-19 infection in an immunosuppressed IEI patient who had recently received anti-CD20 (rituximab) and was not under IgRT. The only treatments available in the first phase of the pandemic, such as steroids, remdesivir, and CP, were used multiple times and in combination, with an overall good response and no adverse effects.

PATIENT 2.

**Figure 2:**
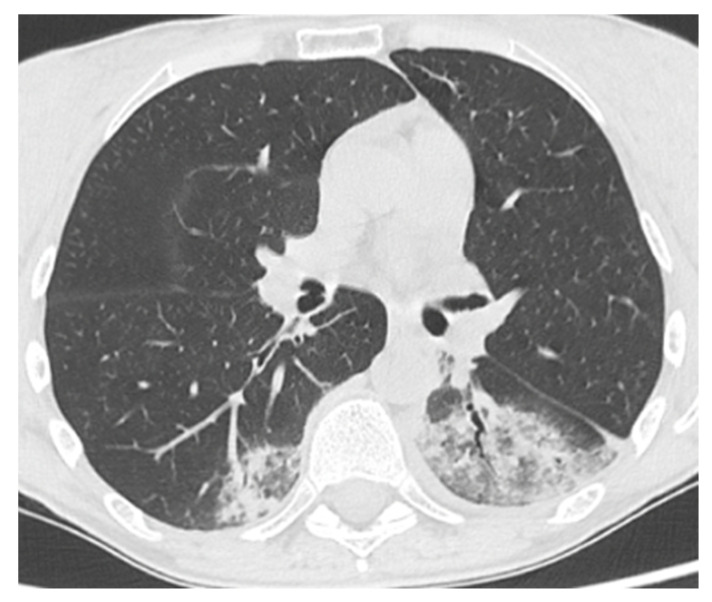
Patient 2: Chest CT scan at time of hospital admission for COVID-19 pneumonia, showing ground-glass opacities with inner foci of consolidation in both lower lobes.

A 50-year-old female patient affected by CVID, complicated by splenomegaly and bronchiectasis in IgRT (SCIG 417 mg/kg/month). The patient had a history of sinopulmonary infections and recurrent Herpes simplex-1 infections with right ocular and labial involvement. On 28 March 2021 (during the Delta wave), she presented with a fever and a cough for a week. For this reason, the GP prescribed antibiotics (amoxicillin/clavulanate) and prednisone 25 mg/day. Because of the persistence of her symptoms, a molecular NPS for SARS-CoV-2 was performed, which tested positive. She was not yet vaccinated (the vaccination campaign had just started in Italy). Due to immunodeficiency, she was eligible for monoclonal antibody therapy, and thus, on 30 March, she was admitted to the infectious disease outpatient clinic. The general examination was unremarkable, except for a fever, but a chest CT scan showed pulmonary bilateral ground-glass opacities and areas of consolidation (Figure 2). On the same day, she received bamlanivimab 700 mg IV. The infusion was well tolerated, and the patient was discharged. On 2 April, the NPS tested negative. After more than two weeks of well-being, the patient experienced a recurrence of a fever of up to 40 °C. On 20 April, she tested positive again for SARS-CoV-2. After 3 days, because of the persistence of her symptoms, she was admitted again to the Infectious Disease Unit. The general examination was again unremarkable, as were her vitals, except for a fever (T 38.4 °C) and mild tachycardia. The blood exams showed mild anemia and a slightly increased D-Dimer (649 ng/mL FEU). A contrast-enhanced chest CT scan excluded pulmonary embolism and showed new pulmonary consolidations and regression of the previously reported consolidations. The microbiological testing was negative, except for SARS-CoV-2 RNA detection in plasma by RT-PCR. Thus, the patient was treated with CP plus remdesivir (5-day course at a dose of 200 mg IV on the first day and 100 mg on the remaining days). The latter was discontinued on the fifth day due to a transaminase elevation of approximately 6–7-fold the normal upper limit (AST 205 U/L and ALT 206 U/L). The therapy resulted in a rapid resolution of the fever. During the second day of hospitalization, nasal vesicles and right conjunctivitis occurred. A vesicle swab tested positive for HSV-1 DNA, and treatment with acyclovir was started accordingly. She was discharged on the seventh day with a SARS-CoV-2-negative NPS. Consecutive NPS tests performed periodically after discharge remained negative. Overall, the patient remained positive for a total of 26 days. At the follow-up visit, the patient showed resolution of herpetic conjunctivitis and normalization of the transaminases. In the following months, the result of the genetic next-generation sequencing (NGS) panel tested positive for a pathogenetic variant of NFKB2 (c.2593G > A; p.Asp865Gly).

In summary, this report shows a patient with NFKB2 haploinsufficiency with a CVID-like phenotype complicated by recurrent Herpes simplex-1 infections who experienced COVID-19 pneumonia. She was treated with mAb with antigenic NPS negativization. However, after more than two weeks, she again presented with a fever and pneumonia concomitant with SARS-CoV-2 RNAemia. Thus, she was treated successfully with CP and remdesivir, although she probably experienced a transient drug-induced liver injury.

PATIENT 3.

A 39-year-old patient affected by XLA (BTK c.994C > T; Arg288Trp), concurrent familial hypertrophic cardiomyopathy diagnosed in early childhood, atrial fibrillation on DOACs, and obstructive renal disease due to nephrolithiasis. He was on IgRT (SCIG 500 mg/kg/month). He repeatedly refused vaccination and got infected with SARS-CoV-2 on 15 August 2021. Twenty days after the infection, he was hospitalized due to the sudden development of respiratory failure in COVID-19 interstitial pneumonia. At admission, he was treated with casirivimab/imdevimab (1200/1200 mg IV), IV steroids, and HFNC. After 10 days of hospitalization, the patient’s condition further deteriorated, and SARS-CoV-2 blood viremia was detected. Thus, he was treated with a course of IV sotrovimab (500 mg in a single infusion) associated with a seven-day course of IV remdesivir (200 mg loading dose, 100 mg maintenance dose). No adverse effects were observed during the treatment. Ten days later, the patient’s condition improved.

On day 14, the blood SARS-CoV-2 RNA turned out negative, as did the RT-PCR on NPS. Oxygen supplementation was completely weaned off on day 15. A few months after he was discharged, in January 2022, the first dose of the BNT162b2 vaccine was administered. The patient was reinfected in June 2022, presenting with a fever and an influenza-like illness for about 3 days and a total of 14 days of NPS positivity. In this case, he did not receive any specific SARS-CoV-2 treatment.

This case shows an IEI patient effectively treated with a combination of antiviral therapy and mAbs against SARS-CoV-2 that was well tolerated, as has been reported in other case series [13]. Moreover, it confirms the well-known protective effect of vaccination (one dose plus previous infection) in cases of reinfection.

PATIENT 4.

A 49-year-old male patient affected by Activated PI3K delta syndrome (APDS1 with mutation in PIK3CD c.1573G > A; p. Glu525lys) with a history of bronchiectasis, chronic obstructive pulmonary disease (COPD), and EBV-related large B cell NHL, treated with R-CHOP regimen (Rituximab, Cyclophosphamide, Hydroxydanorubicin, Vincristine, Prednisone) and substernal radiotherapy. The patient was on IgRT with SCIG (400 mg/kg/month). He was vaccinated with 3 doses of the BNT162b2 vaccine. In the first half of March 2022, the patient was diagnosed as having a relapse of NHL. The therapy was not started because of the onset of rhinitis, fever, fatigue, and dyspnea. An NPS test was positive for SARS-CoV-2 on 23 March 2022. On 27 March, he was treated with sotrovimab 500 mg in a single intravenous infusion as an outpatient. During the infusion, he had a fever up to 40 °C, and the infusion was stopped. Blood samples showed leukopenia (WBC 120 cell/mm³) with neutropenia (50 cell/mm³) and cultures were positive for Staphylococcus hominis. A chest CT scan showed tree-in-bud areas, peribronchial thickening, and bilateral multiple solid nodules. The patient was admitted to the infectious Disease Department in contact isolation due to the persistence of severe leukopenia. A treatment of filgrastim was started with a rise in neutrophils up to 2000 cells/mm^3^. A bronchoalveolar lavage tested positive for extensively drug-resistant Acinetobacter Baumannii. During hospitalization, he was treated with steroids, colistin, and broad-spectrum antibiotics (meropenem, cefepime, and daptomycin). Due to the worsening of the lung disease and persistence of SARS-CoV-2 positivity, the patient was treated with remdesivir a week after sotrovimab, which improved his clinical condition. He was discharged after 28 days of hospitalization, although his NPS was still positive for SARS-CoV-2.

**Figure 3:**
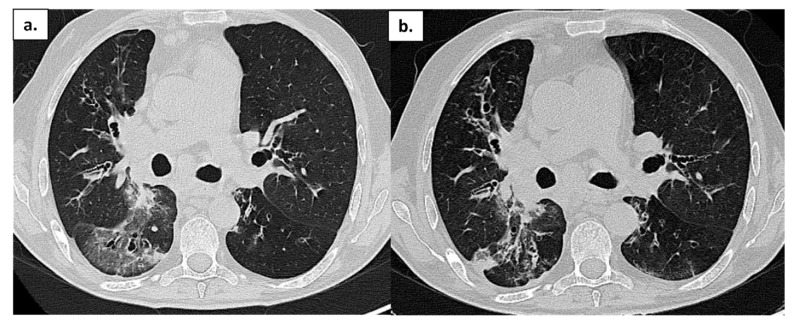
Patient 4: Chest CT in a patient with APDS-1 at onset of COVID-19 pneumonia (**a**) and after treatment (**b**); ground-glass opacities are reduced, but some areas of consolidation are still present.

One month after discharge, the patient’s clinical condition worsened again with the sudden onset of fever and respiratory failure. A NPS tested positive again. He was admitted to the Internal Medicine Department and treated with broad-spectrum antibiotic therapy and high-flow oxygen therapy. A chest CT scan showed an increase in extension of bronchial thickening with confluent aspect and bilateral pleural effusion. On 29 May, the patient experienced acute hypercapnic respiratory failure followed by cardiac arrest. Resuscitation maneuvers were needed to stabilize him. A new CT scan showed extensive ground-glass opacities and some areas of consolidation (Figure 3a). Because of his clinical conditions and persistence of NPS positivity, the patient was treated with CP. Post-treatment control CT scans documented a modest reduction of the parenchymal abnormalities (Figure 3b). On 10 June 2022, he died after a second episode of cardiac arrest due to the onset of pericarditis with cardiac tamponade.

He never tested negative for the control NPS, with an overall duration of positivity of 79 days. He had no adverse effects after both the antiviral and monoclonal therapies for SARS-CoV-2. The bacterial superinfections and the delayed appearance of ground-glass pneumonia are the most frequent complications of immuno-hematological patients with long viral shedding.

PATIENT 5.

**Figure 4:**
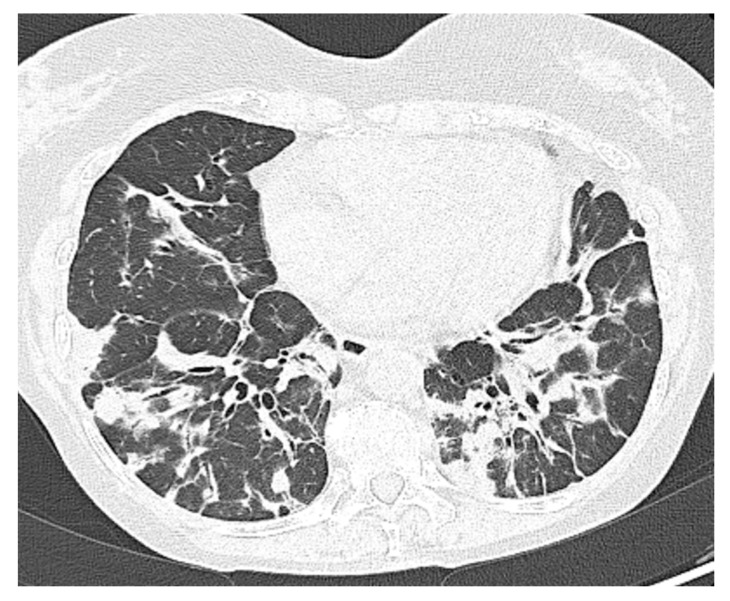
Patient 5: Peribronchial and subpleural irregular consolidations associated with mild architectural distortions resembling organizing pneumonia.

A 69-year-old female patient affected by CVID complicated by GLILD, recurrent lung infections including aspergillosis, Sjögren’s syndrome, and chronic kidney failure. She was vaccinated with 3 doses of BNT162b2 and was under IgRT with SCIG (400 mg/kg/month). Due to the history of recurrent bacterial infections, on 31 March 2022, the patient underwent diagnostic bronchoscopy with a positive bronchoalveolar lavage for Klebsiella pneumoniae carbapenemase-producer (KPC). For this reason, a scheduled hospitalization was organized. Suddenly, on 3 April, she reported the first symptoms of the SARS-CoV-2 infection (fever with chills, general malaise, and lower back pain) and a positive NPS. The patient was thus admitted in advance to the ED. The blood tests showed a rise in CRP, ESR, LDH, and D-Dimer, whereas antigenic NPS had a cut-off index (COI) of 138.70. A chest HRCT was negative for ground-glass opacifications and confirmed the bi-basal lung consolidation and bronchiectasis already known in the patient’s history (Figure 4). During the hospital admission, the patient received IVIG for a sudden onset of fever, lumbar pain, and hypotension. Thus, the IVIG infusion was stopped, and she was accordingly treated with steroids, acetaminophen, and antihistamines. Thereafter, she did not receive IVIG or SCIG anymore during her hospital stay due to concerns about further adverse reactions and IgG levels dropping to 200 mg/dL. During the first days of admission, she received a short-course protocol of IV remdesivir (200 mg on the first day as a loading dose and 100 mg for the two following days). Later on, she needed increasing oxygen supplementation and subsequently received an IV infusion of casirivimab/imdevimab 1200/1200 mg. In the following days, the patient reported a progressive improvement in her clinical condition. On 16 April, she was discharged with a still-positive NPS. On day 52, she tested negative for SARS-CoV-2.

The patient was treated with a combination of antivirals and mAbs for SARS-CoV-2 without any adverse effects. The decision to treat with combination therapy for SARS-CoV-2 was related to the lung involvement and the high risk of superinfection. Of note, the lack of IgRT led to very low IgG levels. The positivity lasted 52 days.

## 4. Discussion

We described a case series of 5 adult IEI patients (2 CVID, 1 NFKB2 haploinsufficiency, 1 APDS1, and 1 XLA) affected by moderate to severe SARS-CoV-2 infection with prolonged positivity during the whole pandemic, ranging from February 2020 until July 2022. All patients received multiple lines of specific treatments against SARS-CoV-2. All patients were hospitalized. One patient died due to cardiac arrest. Interestingly, we observed moderate/severe disease with all the principal VOCs, including Omicron. The clinical data of patients are summarized in Table 1.

It has already been reported that patients with primary antibody deficiencies, such as XLA and CVID, especially those with a low B-cell percentage or patients who have been treated with anti-CD20, present an increased risk for severe viral infections and long viral shedding [14]. Low B-cell percentages are associated with reduced IgA and IgM serum levels, which have a protective role in viral infections [14,15]. In the literature, it is reported that hospitalized CVID patients with COVID-19 presented lower B cell percentages [5,10]. All five patients here presented a low B-cell count (Table 1).

In the literature, the clinical and immunological evaluation of immunodeficient patients that have experienced SARS-CoV-2 infection (or the cross-reactivity of their response to other endemic coronaviruses) has been reported, and in these patients, a robust and specific T-cell response was observed [16,17], even in the context of prolonged viral shedding. Steiner et al. showed in their case series that a relevant negative prognostic factor is the lack of humoral response to SARS-CoV-2 [18].

Several studies have highlighted the risk of prolonged infection in CVID patients [19]. In addition to their primary immunodeficiencies, some of the patients here described presented additional risk factors for severe COVID-19, similar to other reports [20]. In particular, two patients suffered from chronic kidney disease (CKD), and one of them had atrial fibrillation and hypertrophic cardiomyopathy. Moreover, our CVID patients presented CVID-related complications: one had chronic lung damage (bronchiectasis and GLILD) and one had immunodysregulation (RA). Of note, one patient had been recently treated with RTX for RA. The patient with XLA did not present any significant immune-related complications but had multiple cardiovascular risk factors for a severe course of COVID-19. The patient with the NFKB2 mutation had bronchiectasis, splenomegaly, and recurrent HSV-1 reactivation. The patient with APDS1 presented with bronchiectasis, COPD, and a history of EBV-related NHL. Severe cases of COVID-19 have been described for both conditions [21,22,23,24]. At the time of the infection, all but one patient were on IgRT; moreover, one patient could not continue the IgRT during the SARS-CoV-2 infection. Both patients who were not receiving IgRT reached low levels of IgG during the infection course. The absence of IgRT in hypogammaglobulinemic patients has already been associated with a severe disease course [25], highlighting the importance of maintaining (or starting) IgRT during SARS-CoV-2 infection. Moreover, in the latest phases of the pandemic, IgRT products have been shown to contain IgG against SARS-CoV-2, although the clinical impact is unclear [26].

Regarding the specific anti-SARS-CoV-2 treatments, they have been developed to treat patients with risk factors for severe SARS-CoV-2 disease (such as age, cardiovascular comorbidity, diabetes, and previous lung conditions), including IEI patients with complicated phenotypes and cardiovascular comorbidities [10,27].

In our case series, combination therapy and multiple lines of treatment were used (mAbs, antiviral, CP, mAbs + antiviral, CP + antiviral) (Table 1) due to the severity of the infections and/or the prolonged symptomatic diseases with associated complications. Indeed, the long SARS-CoV-2 positivity is associated with a risk of superinfections in CVID patients [10].

Furthermore, prolonged SARS-CoV-2 infection in immunocompromised patients was linked to viral evolution and the possible development of drug resistance [28,29,30].

The use of combinations and multiple lines of therapy has been reported in other case series on immunosuppressed patients with IEI or secondary immunodeficiency [13,31]. In particular, Brown et al. showed that combination therapy including CP or mAbs and remdesivir was more efficient in viral clearance than remdesivir alone [31]. CP has been used for more than a century for treating emerging infectious disease outbreaks, and it was commonly used early in the pandemic in the absence of other therapeutic alternatives. Currently, available trials have shown its efficacy, especially in the early phase of the infection [32,33], but it is now being replaced by mAbs, which generally present a better safety profile [34]. CP has also been used in immunosuppressed patients, with some case series showing its efficacy in patients previously treated with or receiving anti-CD-20 mAbs [35]. In our case series, the CP was used in the late phase of infection as the second- or third-line agent (alone or in combination with remdesivir) with overall good responses and without any adverse effects. We speculate that in our population, the probable impaired antibody response to the virus could explain the efficacy of the CP in the late phases of infection. Furthermore, SARS-CoV-2 blood RT-PCR was performed in two patients after the first line of treatment. A few reports have described the possible negative prognostic role of RNAemia during COVID-19, and this was an element that suggested a treatment with second-line agents considering that SARS-CoV-2 viremia is associated with worse outcomes in hospitalized COVID-19 patients [18,36,37]. In our experience, all therapies were well tolerated except for patient 2, who suffered from probable drug-induced liver injury (DILI). In support of this diagnosis, the patient did not receive any other new drug, and the liver enzymes normalized with remdesivir suspension. The hepatic toxicity of remdesivir has already been described [38].

It is worth noting that none of the patients in this case series had been treated with immunomodulatory drugs to prevent the inflammatory stages of infection (e.g., Anakinra or Tocilizumab), due to the absence of marked elevation of inflammatory markers and possibly due to fear of superinfection in IEI patients. Superinfections have been associated with worse outcomes from SARS-CoV-2 infections [10]. In our cohort, only two patients had been fully vaccinated (with three doses) at the time of infection, mainly due to the date of infection (the vaccination campaign started in January 2021 in Italy) and in one case because of a patient’s refusal. Unfortunately, we did not evaluate the vaccination response in all these patients. However, XLA and CVID patients with complicated phenotypes showed a lower rate of vaccine response compared to healthy controls and mild antibody deficiency [39]. In the case of a lack of vaccine response, it is suggested to use tixagevimab/cilgavimab as a prophylaxis (https://www.ema.europa.eu/en/news/ema-recommends-authorisation-covid-19-medicine-evusheld, last access: 15 February 2023). This treatment does not preclude the use of early therapy with mAbs or antivirals if a breakthrough infection occurs [40].

We highlight that, in our case series, the data collection was retrospective, and the population was heterogeneous due to the different clinical characteristics and the different time periods considered. Thus, it is difficult to generalize the use of similar treatments in the general frail population or in IEI patients in general. However, we described patients with rare conditions and complicated phenotypes, and strong evidence of drug efficacy or best practices cannot always be collected.

## 5. Conclusions

Our case series may support previous studies on the use of combination therapy (and, if necessary, multiple lines of treatment) in adult IEI patients with moderate/severe COVID-19 or prolonged infection. In agreement with a growing number of studies, we believe that the presence of a “complicated” phenotype is a fundamental element to promptly start antiviral or monoclonal therapy and, in cases of failure or prolonged positivity, to use a second or a third therapeutic line for SARS-CoV-2. This is also to avoid complications due to long-term viral positivity, in particular bacterial superinfections. In clinical practice, we highlight that for adult IEI patients, there is still a need to maximize adherence to prophylaxis measures (such as vaccinations and boosters).

## Figures and Tables

**Table 1:** Clinical features of the five patients described.

	Case 1	Case 2	Case 3	Case 4	Case 5
Diagnosis	CVID	NFKB2 (CVID-like)	XLA	APDS1	CVID
Genetic Mutation	Not known	NFKB2 (c.2593G > A; p.Asp865Gly)	BTK (c.994C > T; Arg288Trp)	PI3KCD (c.1573G > A; p.Glu525lys)	Not known
Age	50	50	39	49	69
Sex	F	F	M	M	F
Comorbidity	RA	Splenomegaly, bronchiectasis; recurrent viral infections	Hypertrophic cardiomyopathy; AF; CKD secondary to nephrolithiasis. C	Bronchiectasis; COPD; EBV-related NHL	GLILD; Bronchiectasis; colonization Aspergillus; Sjogren syndrome; CKD
Chronic steroid therapy	no	no	no	no	no
Continuative Immunosuppressant	yes (RTX)	no	no	no	no
Infection period	02/2020	03/2021	08/2021	03/2022	04/2022
Presumptive SARS-CoV-2 variant	Wuhan	Delta	Delta	Omicron	Omicron
Vaccine doses at infection	0	0	0	3 (BNT162b2)	3 (BNT162b2)
	Case 1	Case 2	Case 3	Case 4	Case 5
Diagnosis	CVID	NFKB2 (CVID-like)	XLA	APDS1	CVID
Specific treatment (other than steroids in patients requiring O_2_ therapy)	1st line: remdesivir 2nd line: CP 3rd line: CP + remdesivir	1st line: bamlanivimab 2nd line: CP + remdesivir	1st line: casirivimab/imdevimab 2nd line: sotrovimab + remdesivir	1st line: sotrovimab 2nd line: remdesivir 3rd line: CP	1st line: remdesivr + casirivimab/imdevimab
Hospital admission	yes	yes	yes	yes	yes
Severity according to WHO classification	moderate	moderate	severe	severe	moderate
SARS-CoV-2 related pneumonia	yes	yes	yes	yes	no
O_2_ supplementation therapy needed	LFNC	LFNC	HFNC	HFNC	LFNC
Positivity duration (days)	260	26	14	79 *	52
IgRT	no	yes	yes	yes	discontinued
Comments about IgRT	IgRT started after COVID-19				Interrupted for ADR to IVIG
IgG trough level (mg/dL) during infection course	380	750	800	800	200
IgM (mg/dL)	20	<4	1	178	12
IgA (mg/dL)	6	8	1	1	6
B cell% before infection	0	0	0	0.8	0.4
Bacterial superinfections	yes	no	no	yes	yes
Death	no	no	no	yes	0

Abbreviations: F: female; M: male; RA: rheumatoid arthritis; GLILD: granulomatous and lymphocytic interstitial lung disease; AF: atrial fibrillation; CKD: chronic kidney disease; RTX: rituximab; EVE: everolimus; CsA: cyclosporine; COPD: chronic obstructive pulmonary disease; AIC: autoimmune cytopenia; NHL: non-Hodgkin lymphoma; CP: convalescent plasma; LFNC: low-flow nasal cannula; HFNC: high-flow nasal cannula; IgRT: IgG replacement therapy; ADR: adverse drug reaction. * died before swab negativization.

## Data Availability

Data are available upon reasonable request to the corresponding author.
